# Factors that influence the angular error in active knee angle reproduction tests: A systematic review and meta‐analysis

**DOI:** 10.1002/jeo2.12091

**Published:** 2024-07-24

**Authors:** Juliane Wieber, Leon Müller‐Rahmel, Rüdiger Reer, Robert Rein, Bjoern Braunstein

**Affiliations:** ^1^ Department of Sports and Exercise Medicine, Institute of Human Movement Science University of Hamburg Hamburg Germany; ^2^ Department of Exercise Physiology and Sports Medicine Olympic Training Centre Berlin Berlin Germany; ^3^ Institute of Biomechanics and Orthopaedics German Sport University Cologne Germany; ^4^ Institute of Training Science and Sports Informatics German Sport University Cologne Germany; ^5^ German Research Centre of Elite Sport German Sport University Cologne Germany; ^6^ Centre for Health and Integrative Physiology in Space German Sport University Cologne Germany; ^7^ Institute of Movement and Neurosciences German Sport University Cologne Germany

**Keywords:** ACL, joint position sense, orthopaedics, physiotherapy, proprioception, rehabilitation, return to activity, return to competition, return to play, return to sport

## Abstract

**Purpose:**

The systematic review and meta‐analysis investigated subject‐independent test factors that influence the absolute angle error in active knee angle reproduction tests.

**Methods:**

Five electronic databases were searched to identify relevant studies published before 20 December 2023. Studies were included that were published in either English or German and that investigated joint proprioception in the healthy knee. Included studies were also required to have participants 18–60 years old and free of lower‐limb injury, neurological disorders and diseases affecting joint position sense. Risk of bias was assessed using a Cochrane risk‐of‐bias tool.

**Results:**

Of the 2023 articles identified, 26 studies (1082 participants) were included in the meta‐analysis. The meta‐analysis showed a significant pooled standard mean difference in the absolute angular error for body orientation, direction of movement and fatigue. Active knee angle reproduction tests were found to have a lower absolute angular error when performed in the sitting position compared to the prone position (SMD = −0.56; 95% CI = −1.00 to −0.12). The absolute angular error was found to be greater in cases of knee flexion compared to knee extension (SMD = 0.71; 95% CI = 0.18–1.24). General and local muscle fatigue were found to result in a higher absolute angular error (SMD = 1.39; 95% CI = 1.04–1.75).

**Conclusion:**

Hence, fatigue, body orientation and direction of movement influence the extent of the absolute angular error in active knee angle reproduction tests. Practitioners should be aware that the test conditions and the patient's level of fatigue can affect the results of such tests and that directly comparing results obtained using different test protocols may not be appropriate. The test protocol should be well documented and applied consistently in the clinical setting.

**Level of Evidence:**

Level III, systematic review with meta‐analysis.

**Abbreviations**
AAE: absolute angular error
DOM: direction of movement
ICC: intraclass correlation coefficient
IEEE: Institute of Electrical and Electronics Engineers library
JPS: joint position sense
ROM: range of motion
RPE: rating of perceived exhaustion
SAE: signed angular error
SD: standard deviation
SEM: standard error of measurement
SMD: standard mean difference

## INTRODUCTION

Proprioception is a complex, multi‐modal construct that is not yet fully understood. Sherrington was among the first to use the term ‘proprioception’, describing it as ‘a character of the stimulations occurring in the deep field of receptors, which are traceable to actions of the organism itself [51]. Grob et al. described proprioception as the sum of kinesthesia and joint position sense (JPS) [16]. Kinesthesia is defined as the awareness of joint movement and is dynamic in nature, while JPS is defined as the awareness of the position of a joint in space and is a static phenomenon [16]. Many receptors cooperate to provide information about body position and movement in space, and they are part of three body systems: the vestibular, visual and somatosensory systems. The latter is responsible for the perception of touch, movement and body segment position [17, 29] and utilizes different types of mechanoreceptors to integrate different sensory perceptions. The two primary types of proprioceptors responsible for JPS are muscle spindles and Golgi tendon organs. Muscle spindles are stretch receptors embedded within muscle fibres that detect changes in muscle length and the rate of change. They provide information about muscle stretch and contraction, which is used to determine the position of the body and the direction of movement (DOM; concentric vs. eccentric movement) [6, 19, 40, 41].

The mechanoreceptors constantly provide proprioceptive information to the motor cortex to ensure adequate movement control [29, 35, 47]. Proprioception is an important aspect of knee stability and thus plays an indispensable role in an individual being able to undertake daily activities and physical exercise. In active knee angle reproduction tests, participants are asked to replicate the position of the target knee using their unaffected or contralateral limb. The outcome of these tests is most often absolute angle error (AAE) or signed angle error (SAE) [19]. These types of joint position reproduction tests offer efficiency and the ability to explore hemispheric asymmetries in sensorimotor abilities [13]. However, the diversity of the methods used for proprioceptive assessment has prevented the identification and implementation of standardized and broadly applicable protocols for reliably detecting differences in JPS [15, 42, 44], and low test validity is an issue. This situation may have arisen due to a lack of understanding of the factors that impact the test results. While it is clear that test results may vary with the protocols and equipment used, which are highly dependent on the clinical setting and practitioners' preferences [19], factors that influence proprioceptive perception and any resulting AAE remain to be determined. Hence, before the results from different active knee angle reproduction tests can be reliably compared, the factors that influence the AAE must be identified. Therefore, the aim of this systematic review was to identify subject‐independent test factors that affect the AAE in active knee angle reproduction tests.

## METHODS

### Protocol and registration

Before starting the study, it was registered in the PROSPERO International Prospective Register of Systematic Reviews (registration number: CRD42023333162). The systematic review and meta‐analysis were performed orientated on the Guidelines for Meta‐Analysis from the Cochrane Collaboration (Version 6.4, 2023) [7, 21]. However, contrary to the guidelines, a librarian was not involved, and grey literature was not included. As shown in Table 1, the PICO strategy was used to define the population (P), intervention (I), comparison (C) and outcomes (O) of the included studies to address the following research question: Which subject‐independent test factors influence the AAE in active knee angle reproduction tests? The study followed the Preferred Reporting Items for Systematic Reviews and Meta‐Analysis (PRISMA) guidelines for designing and reporting systematic reviews [37].

**Table 1:** PICO strategy in accordance with the Cochrane Collaboration [21] regarding the research question: Which subject‐independent test factors influence the absolute angular error in active knee angle reproduction tests?

Criteria	Description
**P**opulation	Healthy humans; age 18–60 years
**I**ntervention	Proprioceptive assessment via active knee angle reproduction test
**C**ontrol Conditions	Different active knee angle reproduction test protocols with subject‐independent influencing factors
**O**utcome	Absolute knee angle reproduction error [°]

### Search strategy

Five electronic databases (PubMed, the Institute of Electrical and Electronics Engineers [IEEE] library, SPORTDiscus, Web of Science and the Cochrane Library) were searched for articles published before 20 December 2023. To achieve high sensitivity and minimize the chance of missing relevant articles, a broad search strategy was used. To avoid overlooking any articles, none of the utilized MeSH terms were further narrowed down (see Supporting Information S5 for detailed information on the search strategy).

### Eligibility criteria

**Figure 1:**
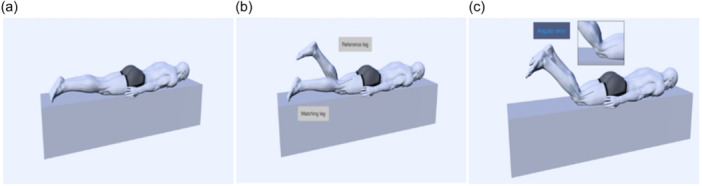
Active knee angle reproduction test in the prone position starting from extension: (a) Start position; (b) Reference leg with target angle flexed; (c) End position with the test limb matching the target angle; Angular error = target knee angle − matching knee angle.

**Figure 2:**
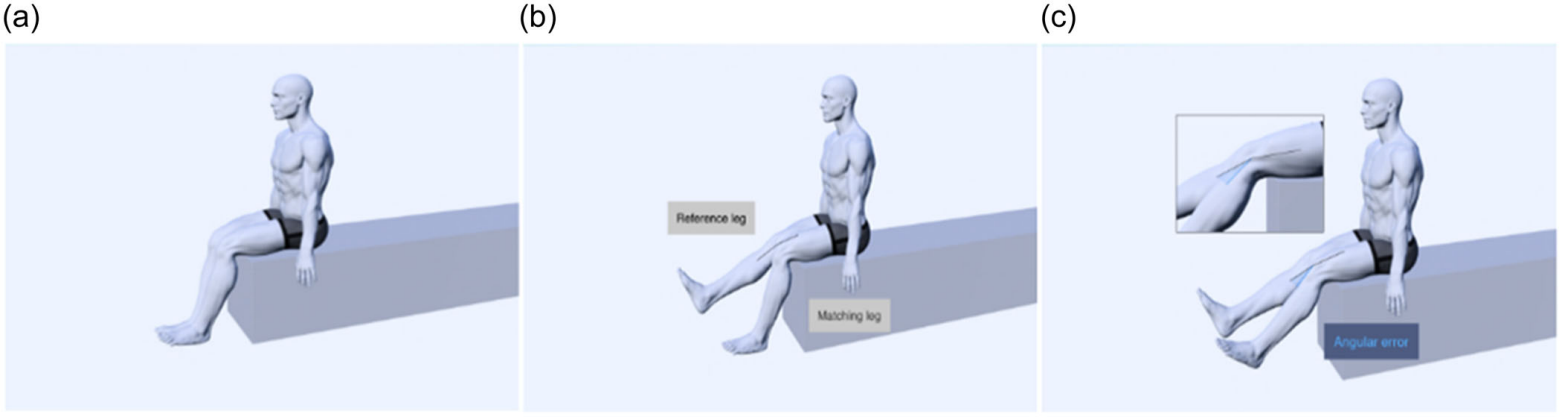
Active knee angle reproduction test in the sitting position starting from flexion: (a) Start position; (b) Reference leg with target angle flexed; (c) End position with the test limb matching the target angle; Angular error = target knee angle − matching knee angle.

For inclusion in this study, articles were required to be peer‐reviewed, published in either English or German, and described studies that investigated joint proprioception in the healthy knee. To be included in our review and meta‐analysis, the described studies had to have participants aged 18–60 years old who were free of lower‐limb injury, neurological disorders and disease affecting JPS, since morbidities such as osteoarthritis, hypermobility syndrome and spasticity influence proprioceptive acuity [2, 22, 54]. Proprioception had to be assessed via an active knee angle reproduction test in a non‐weight‐bearing and open‐kinetic chain position due to gravitational and inertial effects on the sense of proprioception. An active knee angle reproduction test was defined as a test in which the participant had to actively reproduce a given angle with the aim of achieving the smallest possible deviation between the target angle and the reproduced angle of the ipsilateral leg. The target angle had to be chosen by the examiner, and the lower limb had to be moved passively to the target angle (Figures 1 and 2). The subject had to remain in this position for 3–5 s to memorize the angle. Studies with retention times >5 s were excluded. The different factors influencing the active JPS had to be subject‐independent (e.g., not age, gender or level of fitness), and the test had to be completed without the use of aids (e.g., sleeves, braces or tape). The test had to be performed without visual feedback, and the AAE (measured in degrees) had to be an outcome parameter. The AAE has been shown to be more reliable and valid than the SAE in practical settings [34, 42]. Interventions (e.g. playing a match or warming up) that were conducted >1 h before testing were not considered acute. Studies that included knee angle reproduction tests were conducted with additional resistance to create weight‐bearing conditions or knee support (e.g., braces and taping) were excluded, as they did not measure isolated knee JPS under normal conditions [55]. For inclusion in the meta‐analysis, studies had to clearly state the absolute value of the AAE and the standard deviation (SD) or standard error of measurement (SEM). If the SEM was reported, it was converted into the SD (Equation 1).

(1) SD=√SEM·N

### Data collection

After the removal of duplicate articles, two researchers (JW and LMR) independently screened the titles and abstracts using the Rayyan web application [36]. Two researchers (JW and LMR) assessed all potentially eligible full‐text articles to confirm their eligibility. Kappa (*κ*) values were used to assess the inter‐reviewer agreement for the article screening and interpreted as follows: ≤0 no agreement, 0.01–0.20 no to slight, 0.21–0.40 fair agreement, 0.41–0.60 moderate agreement, 0.61–0.80 substantial agreement and 0.81–1.00 almost perfect agreement [28, 31]. Inconsistencies and disagreements were discussed by the researchers and a consensus was reached with the input of a third researcher (BB). Studies were only included when all the authors reached a consensus. If insufficient data were reported, the corresponding author was contacted to request missing data. If the corresponding author did not reply, two reminders were sent at least 12 weeks apart. If there was no response after two reminders or if the corresponding author was not able to provide the requested data, the data were denoted as ‘not available/not retrieved’. All studies that conformed to the PICO strategy met the inclusion criteria, and showed a relevant connection to the research question were included in the systematic review.

### Data extraction

Two authors (JW and LMR) performed the data extraction using standardized data extraction forms found on the Cochrane RevMan Web platform (Version: 7.1.2) [21]. Data on the following parameters were extracted: the publication's details, the number of subjects and their age and level of fitness, the test protocol (position, DOM, target angles and trial repetitions) and the AAE (mean ± SD) for the compared test conditions (see Supporting Information S4).

### Meta‐analysis

The meta‐analysis was conducted using random‐effects models with the inverse variance method, utilizing the RevMan Web application 2023 (Version: 7.1.2) [7]. Standard mean difference (SMD) was employed as the effect measure, while the alpha (*α*) level was set to 0.05. Subgroup analyses were performed to separately assess the influence of subject‐independent factors that were observed in two or more studies using the same test procedure. The reliability and validity of the test procedures were evaluated based on the SEM and intraclass correlation coefficient (ICC). The interpretation of the ICC values adhered to the guideline established by Koo and Li [26].

### Risk of bias assessment

The quality assessment of the included studies was performed by two reviewers who used the risk‐of‐bias analysis tool (ROB 2.0) developed by the Cochrane Collaboration [7, 21]. *κ* values were used to assess the inter‐reviewer agreement for the risk of bias assessment and interpreted as stated earlier. To ensure accuracy, inconsistencies and disagreements were discussed by the researchers, and a consensus was reached with the input of a third researcher (BB). In the risk of bias assessment, a ‘+’ symbol represented low risk, a ‘−’ symbol indicated high risk and a ‘?’ symbol denoted some concerns.

## RESULTS

**Figure 3:**
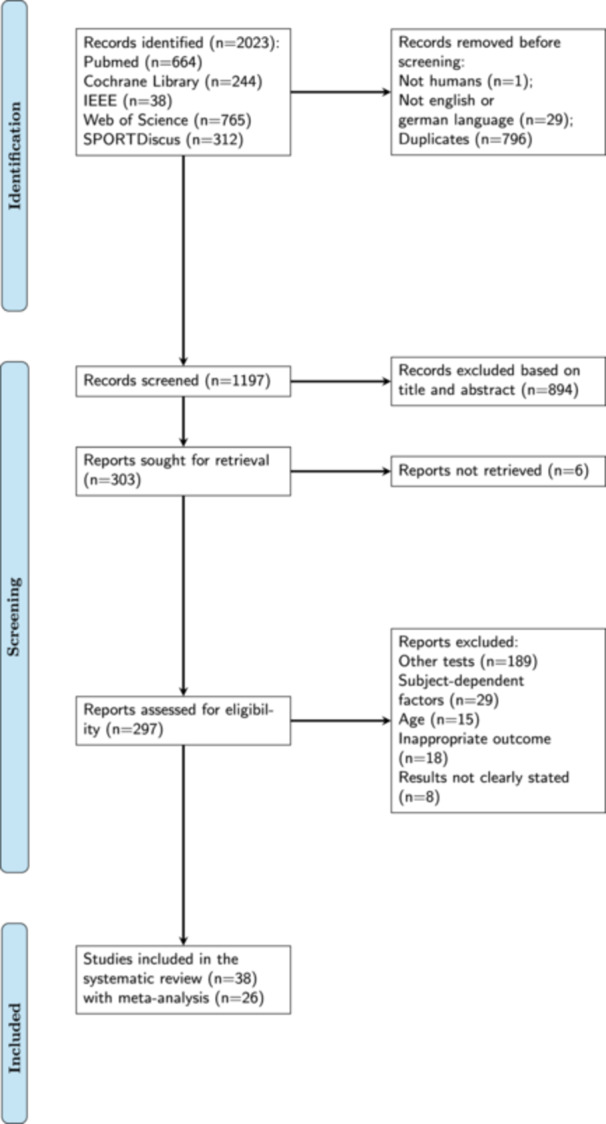
PRISMA flow chart of the study selection process according to the guidelines of the Cochrane Collaboration [21, 37]. PRISMA, Preferred Reporting Items for Systematic Reviews and Meta‐Analysis.

A total of 38 studies were initially identified for potential inclusion in the systematic review (Cohen's *κ* = 0.86; 95% confidence interval [CI] = 0.85–0.87), of which 26 met the inclusion criteria for the meta‐analysis (Cohen's *κ* = 0.94; 95% CI = 0.91–0.96). A flow diagram of the selection process is shown in Figure 3. Six studies were not retrieved due to not being able to download a full source file and the authors not responding to multiple requests. Based on the review of the current literature, the main factors that likely influence the outcome of an active knee angle reproduction test are the body position in which the participant is tested (standing vs. sitting), the direction of the movement (flexion vs. extension) and fatigue (not fatigued vs. fatigued). Protocols designed to induce local fatigue involved interventions that fatigued the knee extensors and/or flexors. Protocols designed to induce general fatigue involved subjects participating in sports matches or running to achieve whole‐body metabolic stress. The comparisons are shown in Table 2. Except for two studies (8%) [33, 43], all the studies (88%) used ≤6 repetitions. In one study (4%), the participants did not undertake multiple trials [20]. The percentage of inter‐rater agreement ranged between 63% and 90% for all seven bias domains. Cohen's kappa was poor for other bias (Cohen's *κ* = 0.09; 95% CI = −0.07 to 0.22) and incomplete outcome data (Cohen's *κ* = 0.05; 95% CI = −0.10 to 0.20), fair for blinding of participants and personnel (Cohen's *κ* = 0.37; 95% CI = 0.19–0.55) and allocation concealment (Cohen's *κ* = 0.32; 95% CI = 0.12–0.50), moderate for selective reporting (Cohen's *κ* = 0.51; 95% CI = 0.39–0.63) and substantial blinding of outcome assessors (Cohen's *κ* = 0.78; 95% CI = 0.69–0.87) and for random sequence generation (Cohen's *κ* = 0.75; 95% CI = 0.66–0.84).

**Table 2:** Allocation of groups for meta‐analysis.

	Groups
Body orientation	Sitting vs. Prone
Direction of movement	Flexion vs. Extension
Fatigue	
Local fatigue	Non‐fatigued vs. Local fatigued
General fatigue	Non‐fatigued vs. General fatigued

*Note*: Local fatigue: involved interventions that fatigued the knee extensors and/or flexors. General fatigue: involved subjects participating in sports matches or running to achieve whole‐body metabolic stress.

### Body orientation

**Figure 4:**
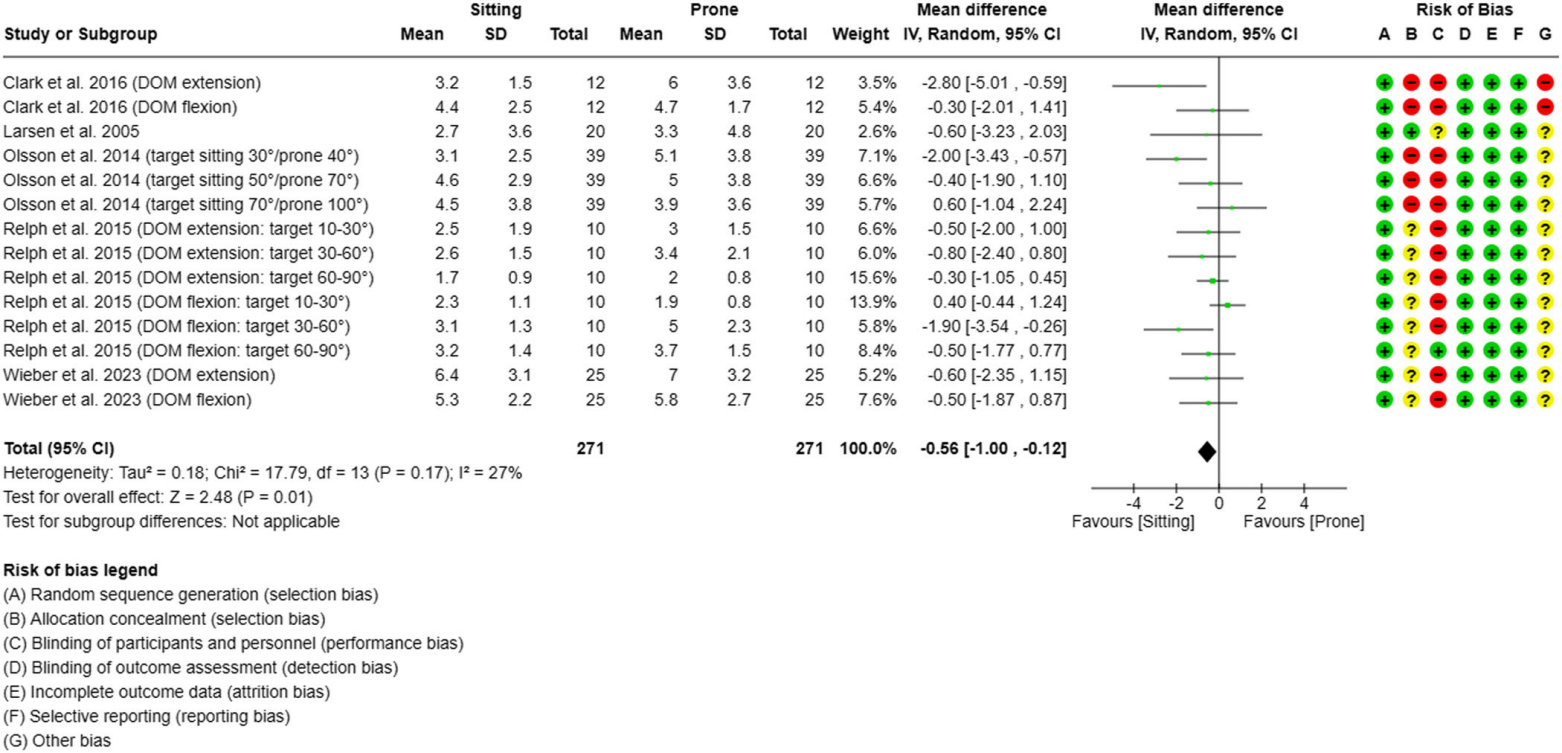
Forest plot of the influence of the subjects' body position on the absolute knee angle reproduction error in active knee angle reproduction tests with subjects tested in the prone or sitting body position. CI, confidence Interval; DOM, direction of movement; IV, inverse variance; SD, standard deviation; target, target angle.

The meta‐analysis indicated that there was a significant difference in the AAE when the participants were tested in the prone position (*n* = 271 participants, 50%) compared to the sitting position (*n* = 271 participants, 50%). However, the heterogeneity was low (*I* ^2^ = 27%) (Figure 4). In the middle of the physiological range of motion (ROM; 30–70° knee flexion), higher errors were noted in the prone position. At the lower (10–30°) and higher (70–100° knee flexion) end of the ROM, the error in the sitting position was higher [34, 42, 59]. Three studies (60%) investigated test–retest reliability and showed ICCs between 0.13 and 0.92 for the seated position and ICCs between 0.17 and 0.90 for the prone position [6, 34, 42]. The SEM values ranged from 0.40° to 1.98° in the seated position and from 0.45° to 3.14° in the prone position [6, 34, 42, 59]. An overview of the overall AAE (mean ± SD) and SMD values is given in Figure 4 (see Supporting Information S1 for detailed study information).

### Direction of movement

**Figure 5:**
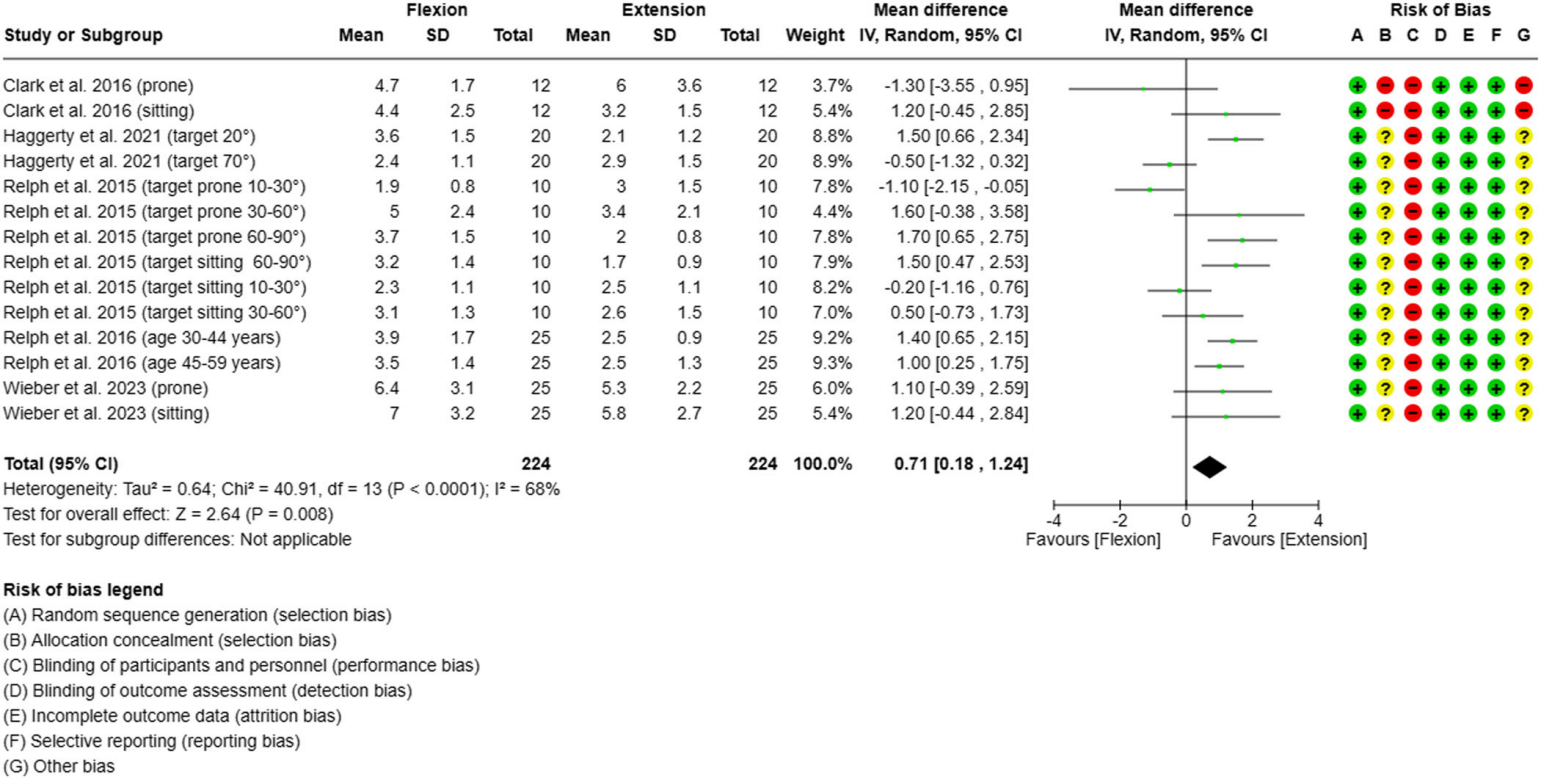
Forest plot of the influence of the direction of movement of the subjects' lower limb on the absolute knee angle reproduction error in active knee angle reproduction tests. CI, confidence interval; IV, inverse variance; SD, standard deviation; target, target angle.

The results of the meta‐analysis indicated that the AAE was significantly greater during knee extension (*n* = 224 participants, 50%) than during knee flexion (*n* = 224 participants, 50%). However, the heterogeneity was moderate (*I* ^2^ = 68%) (Figure 5).

At the lower end of the ROM (10–30°), higher errors were observed during flexion. In the middle and towards the higher end of the ROM (30–90°), the error was higher during extension [42]. One study (20%) demonstrated that the AAE was higher during flexion with a target angle of 20° but higher during extension with a target angle of 70° [18]. Two studies (40%) investigated test–retest reliability and showed poor to good ICCs (0.13–0.87) for knee extension and poor to excellent ICCs (0.03–0.90) for knee flexion [6, 42]. The SEM values ranged from 0.5° to 1.8° for knee extension and from 0.4° to 3.1° for knee flexion [6, 42, 59]. An overview of all the AAE (mean ± SD) and SMD values is given in Figure 5 (see Supporting Information S2 for detailed study information).

### Fatigue

**Figure 6:**
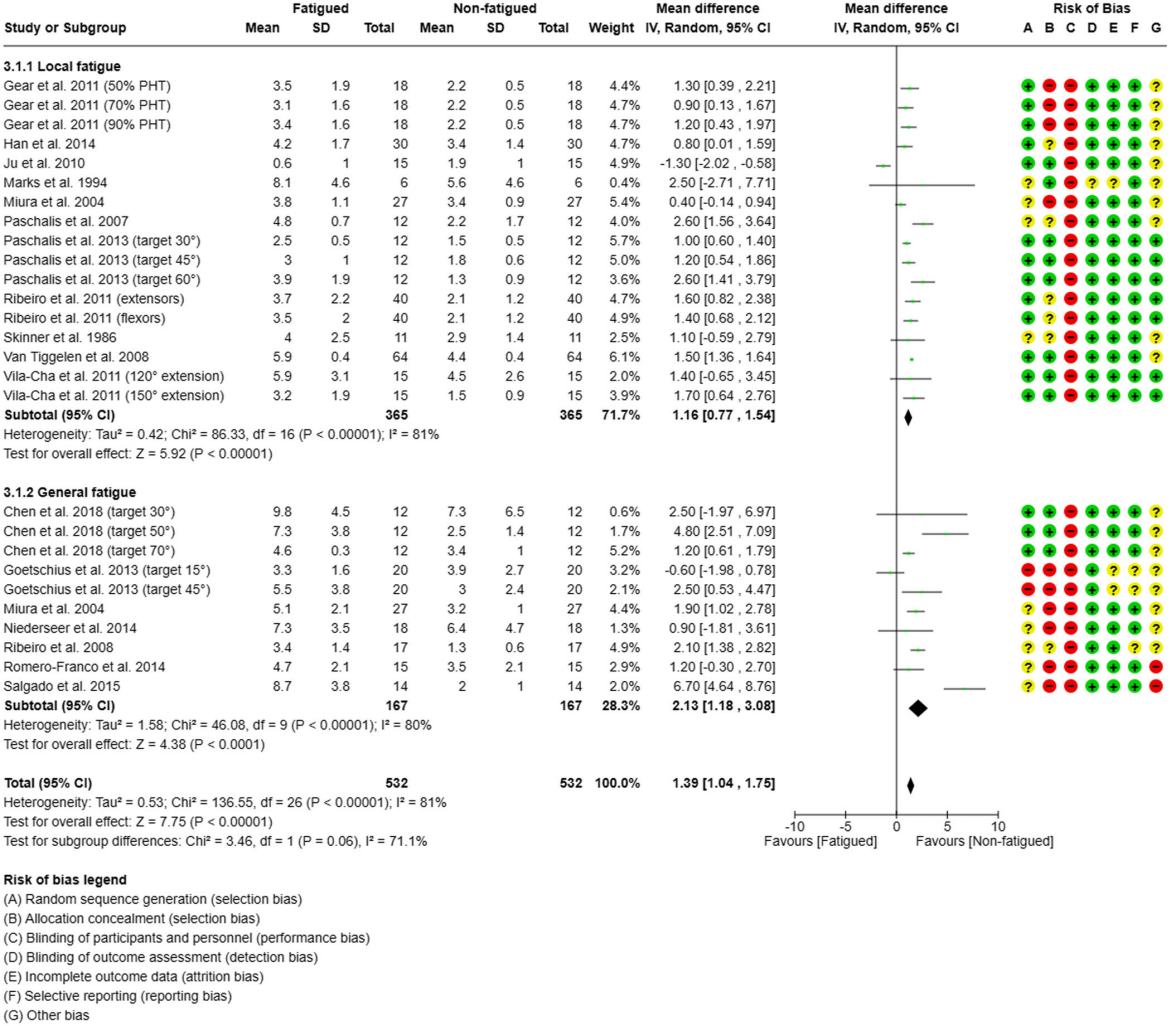
Forest plot of the influence of fatigue on the absolute knee angle reproduction error in active knee angle reproduction tests. Subgroup analyses were performed to assess the influence of fatigue, distinguishing between local fatigue (studies involving protocols that fatigued the knee extensors and/or flexors) and general fatigue (studies that used pre‐post match designs and running/cycling protocols inducing whole‐body metabolic stress). CI, confidence interval; IV, inverse variance; SD, standard deviation; target, target angle.

The meta‐analysis results indicated that the AAE was greater when the subject was fatigued (*n* = 532 participants, 50%) than non‐fatigued (*n* = 532 participants, 50%), with the studies showing high heterogeneity (*I* ^2^ = 81%) (Figure 6). Most of the studies (76%) reported a significant decline in proprioceptive acuity measured in an active knee angle reproduction test [5, 11, 14, 20, 38, 39, 45, 46, 49, 50, 53, 57, 58]. The studies were subdivided into those that involved local and general fatigue. An overview of all the AAE (mean ± SD) and SMD values is given in Figure 6 (see Supporting Information S3 for detailed study information).

#### General fatigue

The subgroup analysis of the effect of general fatigue indicated that the AAE was greater when subjects were fatigued (*n* = 167 participants, 50%) than non‐fatigued (*n* = 167 participants, 50%), with the studies showing high heterogeneity (*I* ^2^ = 81%) (see Figure 6). To induce general fatigue, three studies (43%) used treadmill protocols (incline of 1%–15%) [5, 14, 32]. One of these studies used a treadmill protocol combined with jumping exercises [14]. All these studies showed a significantly greater AAE after the general fatigue interventions were administered [5, 14, 32]. Furthermore, the systematic review included four studies that used field‐based fatigue protocols (57%); in three studies, the subjects participated in matches in pivoting sports [33, 45, 50], and in one study, the subjects undertook a running protocol [49]. The match‐based protocols included playing volleyball [50], football [45] and handball [33] for 60–90 min. In the study that included the subjects playing handball, no significant decline in AAE was observed after the handball match (*p* = 0.56; *d* = 0.13, 95% CI = −0.58 to 0.31), although the subjects were subjectively fatigued (RPE pre‐intervention: 7 ± 1; RPE post‐intervention: 13 ± 2; *p* <0 *.*001) [33]. One field‐based study (25%) investigated the short‐term effects of anaerobic lactic exercise on track‐and‐field athletes and demonstrated a significant increase of 1.2° in the AAE post‐intervention (*p *< 0.001) [49].

#### Local fatigue

The subgroup analysis of the effect of local fatigue indicated that the AAE was greater when subjects were fatigued (*n* = 365 participants, 50%) than non‐fatigued (*n* = 365 participants, 50%), with the studies showing high heterogeneity (*I* ^2^ = 81%) (see Figure 6). Significantly greater AAE values were observed after the local fatigue protocols, except in three studies (23%) [24, 30, 32]. In 10 studies (77%), local fatigue was induced using an isokinetic dynamometer and 15–30 concentric and eccentric hamstring [11, 46, 57] and quadriceps contractions [25, 30, 32, 38, 39, 46, 57, 58]. In one study (7%), local fatigue was induced by having the subjects perform all‐out sprint intervals and included an inclined treadmill protocol (15% incline) [53]. In another study (7%), subjects performed three sets of 10 squats with 30 s of rest between sets [20]. In yet another study (7%), subjects undertook six cycles of inclined treadmill walking (5 min) and jumping exercises (1 min) [14].

The following parameters were variously measured to assess muscle damage and fatigue control: isometric peak torque (>10% decrease), lactate levels in the blood, heart rate [38, 39, 48] and a fatigue index (%) [11, 24, 25, 30, 32, 46, 53, 57]. Three studies (21%) either did not have a fatigue cutoff criterion [20, 58] or subjectively rated perceived exhaustion [14].

## DISCUSSION

The purpose of this systematic review and meta‐analysis was to investigate subject‐independent test factors that influence the AAE in active knee angle reproduction tests. Testing in a seated position and extending the knee were both found to be associated with lower AAE values and more reliable measurements. However, both examined positions and directions of movement showed wide ICC ranges. Furthermore, the target angle and the interaction between the body position and DOM seemed to affect the AAE. Inducing fatigue by means of intensive exhaustion was found to increase the AAE irrespective of the presence of local or general fatigue. Although the overall risk of bias was low for the included studies, the risk of bias for blinding remained present. It is worth noting that participants in these types of studies cannot be blinded in terms of the DOM, their body position or being fatigued. Additionally, there were some concerns about the use of subjective scales, such as the RPE for evaluating fatigue (see Figure 6). Due to the high heterogeneity observed, future research should focus on investigating the multi‐factorial influence of the most important test factors (i.e., body position, DOM, limb dominance and the target angle) on the AAE. Furthermore, given that the accuracy of the measured outcome is of utmost importance in terms of clinically evaluating the success of interventions and rehabilitation progress, future research should also be conducted to optimize measurement reliability. Our specific findings are discussed in more detail below.

### Body orientation

The systematic review revealed that body orientation had a significant effect on the AAE, with the seated position found to be favourable (SMD = −0.56; 95% CI = −1.00 to −0.12). Wieber et al. previously proposed that significant differences in AAEs could result from disparities in body position, particularly the position of the head (upright vs. horizontal) [59]. In addition, variation in tactile feedback from the thigh might have influenced the observed differences in JPS. One of the studies reported that, in a seated position, the primary source of tactile feedback is located on the back of the thigh, whereas in a prone position, the front of the thigh is more likely to provide tactile feedback [56]. Da Silva et al. provided further evidence for this notion and concluded that eccentric‐induced position sense alterations may arise from central and/or peripheral mechanisms depending on the testing position [8]. Regarding measurement reliability, Clark et al. showed that results from seated‐position tests were not reliable, with insufficient ICCs (95% CI) between 0.13 (−0.62 to 0.58) and 0.31 (−0.31 to 0.74) [6]. They demonstrated that prone‐position tests generated moderate to good ICCs (95% CI) between 0.51 (0.01–0.83) and 0.87 (0.61–0.96), depending on the DOM. The SEM values associated with the prone and sitting positions were almost identical [6].

Olsson et al. found that the ICC varied greatly in both types of tests (sitting: ICC = 0.31–0.82 vs. prone: ICC = 0.17–0.75) [34]. These results were not supported by Relph and Herrington, who showed a higher reliability for the sitting position with good to excellent ICCs (0.65–0.90), and the highest ICC value (0.92) recorded in the seated position, while testing the dominant leg in a flexing movement at a range of 60–90° [42].

### Selection of the target angle

Two studies concluded that the most reliable measurement was obtained when the target angle was 60–90° [6, 42]. In the middle of the physiological ROM (30–70°), higher AAE values were reported for the prone position, whereas at the lower (10–30° knee flexion) and higher (70–100° knee flexion) ends of the ROM, the AAE values were higher for the sitting position [24, 30, 41]. Clark et al. determined that selecting angles within the middle of the ROM, specifically around 45°, may lead to reduced activation of capsuloligamentous mechanoreceptors, while preferentially stimulating musculotendinous mechanoreceptors [6]. This approach might also help relax antagonistic muscles by avoiding excessive end‐of‐ROM stretching and minimizing the activation of antagonistic mechanoreceptors [6]. Furthermore, most sports‐specific movement patterns fall in the middle of the ROM [52] and non‐contact knee injuries, especially anterior cruciate ligament injuries, occur when the knee is in the middle of the ROM [27, 44]. This suggests that proprioceptive acuity is influenced by the target angle and is limited in the middle of the ROM.

### Direction of movement

The analysis of the study data revealed that the DOM has a significant effect on the AAE, with higher average AAE values recorded during flexion compared to extension (SMD = −0.71; 95% CI = 0.18–1.24). Gravity has been shown to play an important role when comparing movement directions [60]. The concentric muscular strain on the joint structures is higher and the pressure conditions in the joints are different when moving against the force of gravity. This might also be due to the increased activation of the muscle spindles and the Golgi tendon organ during contraction of the larger concentric quadriceps muscle [43, 59]. Wieber et al. discussed how pre‐tension of the m. quadriceps femoris, depending on the stretching ability, may result in better reproducibility [59]. However, Haggerty et al. reported that young, healthy, active individuals without negative impacts on the afferent system might not benefit from additional spindle traffic from an eccentric‐to‐isometric contraction [18]. They demonstrated that the DOM from extension to flexion led to a significantly greater AAE than the concentric contraction from flexion to extension of the knee joint when the target angle was 20°; however, they could not achieve a similar result when the target angle was 70° [18]. Clark et al. did not draw any conclusions about the DOM due to poor reliability and were not clear about why hamstring‐focused tests demonstrated reliability in contrast to quadriceps‐focused tests [6]. They demonstrated inconclusive poor to good ICCs for extension (ICC; 95% CI; SEM) (sitting: 0.13; −0.62 to 0.58; 1° vs. prone: 0.87; 0.61–0.96; 1°) and flexion (sitting: 0.31; −0.31 to 0.74; 2° vs. prone: 0.51; 0.01–0.83; 1°), whereas reliability was higher in the prone position compared to the seated position [6]. In contrast, Relph and Herrington tested in a seated position and demonstrated good to excellent ICCs for extension (0.51–0.87) but inconclusive ICCs for flexion (0.03–0.90) [42].

### Fatigue

The results of the meta‐analysis demonstrated that fatigue had a significant overall effect on the AAE (SMD = 1.33; 95% CI = 1.23–1.44). The subgroup analysis revealed that both local and general fatigue had a statistically significant effect on AAE as a parameter indicating proprioceptive acuity (see Figure 6). All the studies that investigated the effect of general fatigue demonstrated that there was a significant decline in proprioception. However, Goetschius et al.'s study, in which participants were tested with a challenging 15° angle, did not yield statistically significant results [14]. A possible reason for the decline in AAE after inducing fatigue is that the exercised muscle was perceived to be longer than it actually was, which influenced proprioceptive acuity [12, 38, 39]. This explanation is based on the ‘forward model of motor commands’ proposed by Bays and Wolpert [3]. According to this model, a motor command is used to recreate a reference position, which is then translated into movement. A copy of the motor command is sent to the forward model, which predicts the body position that will be reached. Fatigue‐induced noise, from either the system or the environment, can introduce differences between the actual and perceived body position [3]. Furthermore, it is proposed that fatiguing the lower limbs results in a decreased efferent response and proprioceptive ability and a higher AAE, as the musculature of the thigh has a high percentage of fast‐twitch muscle fibres, which rapidly fatigue during exercise and has greater afferent innervation than slow‐twitch fibres [11]. It was also suggested in another study that warm‐up effects may lead to neuromuscular reflex enhancement and thus cause an improvement in JPS [24]. These warm‐up effects were also noted in another study [50] but were not supported by other studies that used a warm‐up protocol before a fatigue protocol [39, 46, 53]. The level of fatigue and level of fitness were also found to be associated with the AAE, and fitter subjects were observed to be more resilient to fatigue [12, 39]. Even when methods were employed to control the exercise intensity before testing, researchers found that trained athletes tended to exhibit smaller differences between pre‐ and post‐fatigue measurements compared to more sedentary participants [39, 48]. Hence, it might be important to consider the fitness level of each participant when the testing protocol involves exercises that could induce fatigue before proprioceptive testing. Trained athletes seem to have a higher threshold for fatigue and require greater levels of fatigue to demonstrate a decline in proprioceptive acuity. Givoni et al. reported a correlation between mean AAE and a decline in force during the exercise protocol [12]. The included studies with professional athletes were inconclusive, with one study reporting much higher AAE values and broader SD values than other studies; an AAE of 7° was observed after a 90‐min handball match [33]. This might have been due to the participants experiencing a long period of mental stress, which also involved jumping and running. Severe physical exertion tends to impair mental performance, while moderate physical exertion significantly improves it [9]. The protocols included in this review likely involved an intermediate level of physical exertion. Different levels of physical exertion may lead to varying results amongst individuals, indicating a dependence on the level of physical exertion and the fitness level of the participant [9]. It is also possible that pathways unaffected by muscle mechanoreceptors may have been influenced by the protocols used in the studies [32, 46]. Ribeiro et al. found that proprioception was diminished in both eccentric and concentric conditions. Since concentric exercise is expected to result in minimal to no damage or disruption of muscle spindles, diminished proprioception may not be solely attributed to insufficient muscle receptor function but also to metabolic stress (i.e., changes in concentrations of metabolites and inflammatory substances) [46]. It is worth noting that exercise‐related substances, such as lactic acid, arachidonic acid and bradykinin, can also impact afferents from muscle spindles, potentially influencing proprioceptive acuity without directly affecting muscle strength [4, 10, 23].

### Strengths and limitations

This study has several strengths. First, it was based on the Guidelines for Meta‐Analysis from the Cochrane Collaboration (Version 6.3, 2022) [7, 21] and included a structured analysis that adhered to the PRISMA guidelines and PICO strategy [21, 37]. Second, the study protocol was registered in the PROSPERO International Prospective Register of Systematic Reviews (registration number: CRD42023333162), ensuring methodological rigor. Additionally, it employed a best‐evidence synthesis approach, incorporating levels of evidence and risk of bias for all potential risk factors, thus providing a comprehensive overview of the current literature on the main subject‐independent factors influencing AAE in active knee angle reproduction tests. Despite these strengths, there are methodological limitations to consider. First, only studies published in English and German were included, which potentially introduced selection bias. Second, the meta‐analysis revealed that there was considerable heterogeneity amongst the studies examined that investigated the influence of fatigue. This might have been due to the different measuring devices and interventions utilized in the studies. Therefore, the results of this part of the meta‐analysis should be interpreted with caution. Third, the tests were conducted on healthy knees. Therefore, different outcomes may be observed when patients with knee injuries, such as anterior cruciate ligament injuries, are tested in clinical settings. Fourth, only participants aged 18–60 years old were included. Although only studies in which no osteoarthritis was reported were included, it cannot be assumed that people over the age of 60 may still have undetected symptoms of osteoarthritis. Fifth, a librarian was not involved in this study, and grey literature was not included, which is a Cochrane Reviews quality criterion. Finally, this review focused solely on subject‐independent factors, neglecting potential influences such as body mass and menstrual cycle, which have been found to impact proprioception [1, 39].

## CONCLUSION

The findings indicate that fatigue, body orientation and the direction of movement of the lower limb have an impact on the active angular error in active knee angle reproduction tests. Furthermore, these factors may impact each other and are dependent on the target angle. Thus, it is not appropriate to directly compare results obtained using different test protocols. It is also important for practitioners to recognize that altering test conditions can have diverse therapeutic implications, and it is recommended to test in a non‐fatigued situation to avoid a decline in proprioceptive acuity. Finally, the test protocol should be well documented and applied consistently in the clinical setting.

## AUTHOR CONTRIBUTIONS

Juliane Wieber: Conceptualization; methodology; software; investigation; software; visualization; writing—original draft; writing—review and editing; formal analysis; data curation. Leon Müller‐Rahmel: Draft preparation; data curation; writing—review and editing. Robert Rein: Supervision; resources; conceptualization. Rüdiger Reer: Supervision; resources and editing. Bjoern Braunstein: Conceptualization; supervision; visualization; resources; project administration; writing—review and editing.

## CONFLICT OF INTEREST STATEMENT

The authors declare no conflict of interest.

## ETHICS STATEMENT

Due to the study design no ethics statement is required.

## Supporting information

Supporting information.

Supporting information.

Supporting information.

Supporting information.

Supporting information.

## Data Availability

The systematic review with meta‐analysis was preregistered in the PROSPERO International Prospective Register of Systematic Reviews (registration number: CRD42023333162). All data generated or analysed during this study are included in this published article and its supplementary information file.
