# Supplementary figures and images for: Factors that influence the angular error in active knee angle reproduction tests: A systematic review and meta‐analysis
Source: J Exp Orthop. 2024 Jul 24;11(3):e12091. doi: 10.1002/jeo2.12091 (PMC11269366; doi:10.1002/jeo2.12091)

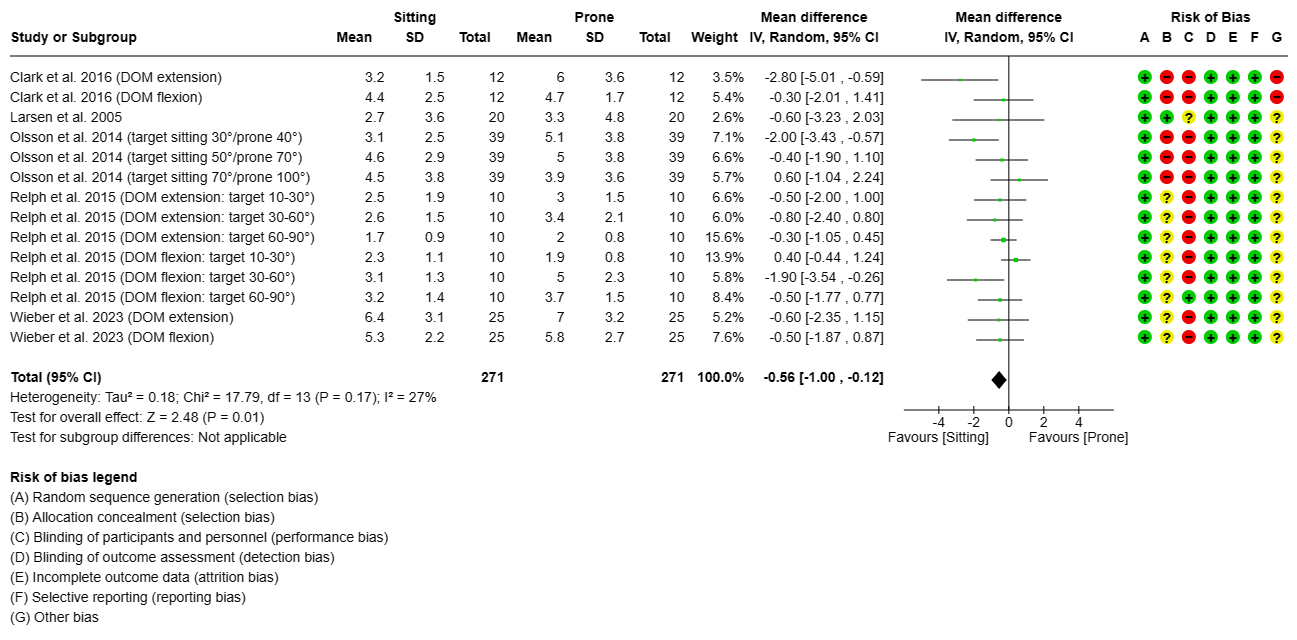

Supplement: Supplementary file 1 — Supporting information. [file JEO2-11-e12091-s001.png]

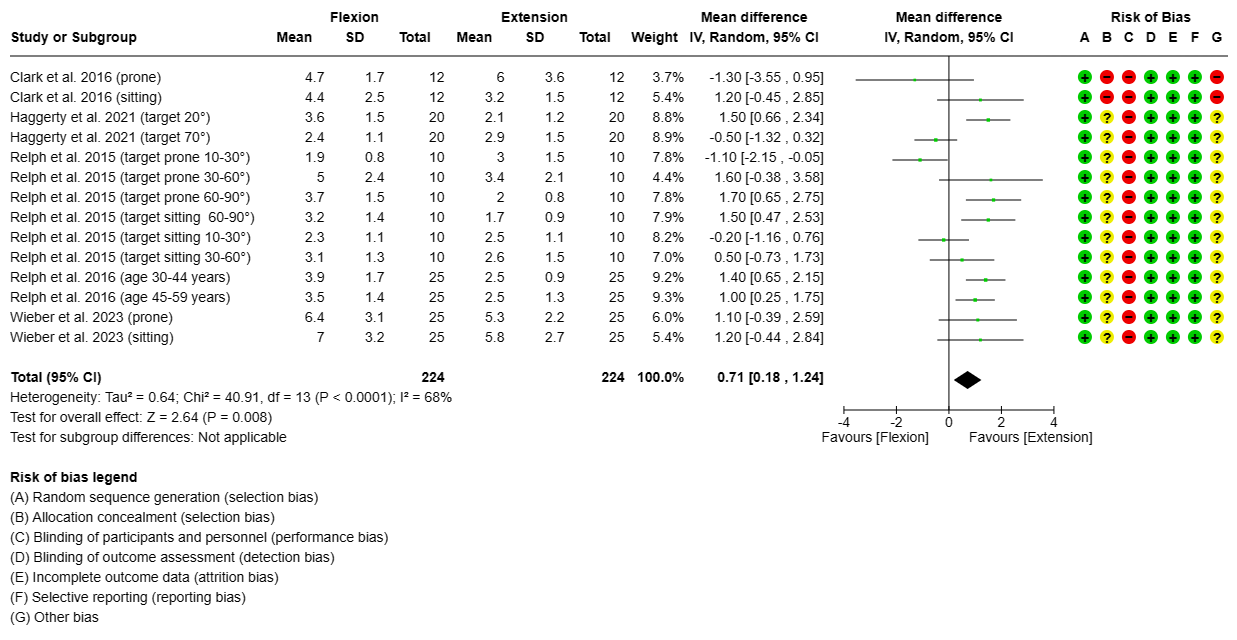

Supplement: Supplementary file 2 — Supporting information. [file JEO2-11-e12091-s005.png]

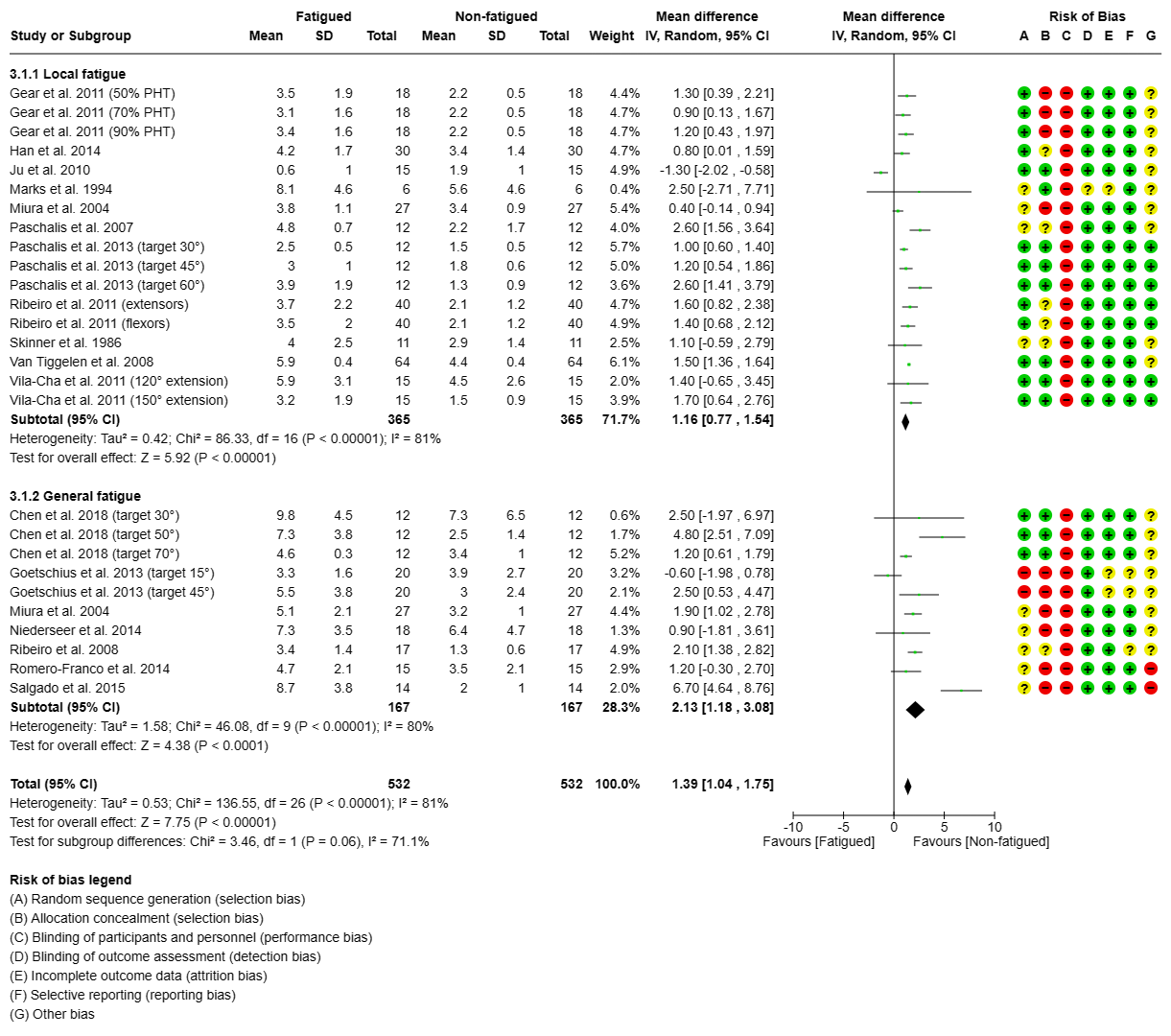

Supplement: Supplementary file 3 — Supporting information. [file JEO2-11-e12091-s003.png]
