# Supplementary material for: Factors that influence the angular error in active knee angle reproduction tests: A systematic review and meta‐analysis
Source: J Exp Orthop. 2024 Jul 24;11(3):e12091. doi: 10.1002/jeo2.12091 (PMC11269366; doi:10.1002/jeo2.12091)
Supplement: Supplementary file 4 — Supporting information. [file JEO2-11-e12091-s002.pdf]

Study	N #	Sex [f/m/b]	Age [yrs.]	Level of Fitness	Body position prone	sitting	Direction of Mov. Extension	Flexion	Target α in [°]	Reps #	Meta [Group]	Intervention
Chen et al. (2018)	12	m	22 ± 1	recreational active	☑	☐	☐	☑	30	5	Fatigue	Treadmill (10 min., 6.4 kmh^{-1}, 1%).
					☑	☐	☐	☑	50	5	Fatigue	Treadmill (10 min., 6.4 kmh^{-1}, 1%).
					☑	☐	☐	☑	70	5	Fatigue	Treadmill (10 min., 6.4 kmh^{-1}, 1%).
Clark et al. (2016)	12	f	26 ± 6	recreational active	☑	☑	☑	☑	45	5	BP & DoM	
Gear et al. (2011)	10	f	19 ± 1	PRO Foot- & Basketball	☐	☑	☑	☐	15-45	3	Fatigue	Hamstrings muscle fatigue: **Phase 1:** (V_α, reps): $90\,°s^{-1}$, 10; $180\,°s^{-1}$, 15; $240\,°s^{-1}$, 20; $300\,°s^{-1}$, 25; 40 s rest. **Phase 2:** $180\,°s^{-1}$ until hamstring PT $<$ 90%, 70%, or 50% PT_{max}, 3 reps.
	08	m	21 ± 1	PRO Foot- & Basketball	☐	☑	☑	☐	15-45	3	Fatigue	Hamstrings muscle fatigue: **Phase 1:** (V_α, reps): $90\,°s^{-1}$, 10; $180\,°s^{-1}$, 15; $240\,°s^{-1}$, 20; $300\,°s^{-1}$, 25; 40 s rest. **Phase 2:** $180\,°s^{-1}$ until hamstring PT $<$ 90%, 70%, or 50% PT_{max}, 3 reps.
Goetschius et al. (2013)	20	b	25 ± 5	recreational active	☐	☑	☑	☐	15	3	Fatigue	Treadmill (5 min., 4.8-5.6 kmh^{-1}, 0%+1%/min to max. 15%) + 1 min squat & lateral jumps); 6 cycles.
					☐	☑	☑	☐	45	3	Fatigue	Treadmill (5 min., 4.8-5.6 kmh^{-1}, 0%+1%/min to max. 15%) + 1 min squat & lateral jumps); 6 cycles.
Haggerty et al. (2021)	20	b	21 ± 2	healthy	☐	☑	☑	☑	20	6	DoM	
									70	6	DoM	
Han et al. (2014)	15	f	21 ± 1	healthy	☐	☑	☑	☐	30-60	1	Fatigue	Quadriceps muscle fatigue: 3 sets; 10 squats; 30 s rest between sets, speed 20 bpm (metronome).
	15	m	22 ± 1	healthy	☐	☑	☑	☐	30-60	1	Fatigue	Quadriceps muscle fatigue: 3 sets; 10 squats; 30 s rest between sets, speed 20 bpm (metronome).
Ju et al. (2010)	15	b	24 ± 1	healthy	☐	☑	☑	☐	10-80	1	Fatigue	Quadriceps muscle fatigue: concentric & eccentric MVC (120°/s & 60 rep).
Kellari et al. (2023)	54	b	21 ± 3	healthy	☐	☑	☑	☐	45	1	Fatigue	Quadriceps muscle fatigue: max. concentric contraction; 20°/s un- til trials ¡50% of the maximal extensors maximal peak torque.
Larsen et al. (2021)	20	b	$25\ (21-31)$	PRO & recreational active	☑	☑	☑	☑	50-70	1	BP	
Marks (1994)	6	f	$18-30$	healthy	☐	☑	☑	☐	45-75	5	Fatigue	Quadriceps muscle fatigue: 20 high-speed reciprocal concentric & eccentric MVC.
Miura et al. (2004)	27	m	$22\ (21-30)$	healthy	☐	☑	☑	☐	10-80	8	Fatigue	MVC (flexion-extension) (120°/s, 60 reps).
											Fatigue	Treadmill (5 min., 10 kmh^{-1}, 10%).
Niederseer et al. (2014)	18	m	22 ± 5	PRO Handball	☐	☑	☑	☐	random	6	Fatigue	90 min handball match.
Olsson et al. (2004)	39		$27\ (18-50)$	healthy	☑	☑	☑	☐	30-40	3	BP	
									50-70	3	BP	
									70-100	3	BP	
Paschalis et al. (2007)	12	f	20 ± 1	untrained	☐	☑	☑	☐	45	4	Fatigue	Isokinetic eccentric MVC (60 $90\,°s^{-1}$, 5 sets, 15 reps, 2 min rest).
Paschalis et al. (2013)	12	f	20 ± 1	untrained	☐	☑	☑	☐	30	4	Fatigue	Isokinetic eccentric MVC (60 $90\,°s^{-1}$, 5 sets, 15 reps, 2 min rest).
									45	4	Fatigue	Isokinetic eccentric MVC (60 $90\,°s^{-1}$, 5 sets, 15 reps, 2 min rest).
									60	4	Fatigue	Isokinetic eccentric MVC (60 $90\,°s^{-1}$, 5 sets, 15 reps, 2 min rest).
Relph & Herrington (2015)	10	f	30 ± 9	Tegener Score: 5 ± 2	☐	☑	☑	☑	10-30	15	BP & DoM	
									30-60	15	BP & DoM	
									60-90	15	BP & DoM	
Relph & Herrington (2016)	25	f	$30-44$	Tegener Score: 5	☐	☑	☑	☑	30-60/60-90	5	DoM	
			$45-59$	Tegener Score: 4	☐	☑	☑	☑	30-60/60-90	5	DoM	
Ribeiro et al. (2008)	17	f	18 ± 2	PRO Volleyball	☐	☑	☑	☐	40-60	3	Fatigue	Volleyball match (90 min match; 25 min warm-up (stretching, passing, setting, attacking, serving exercises)).
Ribeiro et al. (2011)	40	m	22 ± 3	healthy	☐	☑	☑	☐	60	3	Fatigue	MVC concentric/eccentric (extensors & flexors; $180\,°s^{-1}$, 30 reps).
Romero-Franco et al. (2014)	15	b	26 ± 7	PRO Track & Field	☐	☑	☑	☐	40-60	3	Fatigue	**Warm-up**: 30 min (10 min low-speed race (8 km/h), 5 min dynamic stretching,15 min technical running exercises); **Lactic Exercise**: 2 groups of 2 series of 300 m performed at 90–92%, rest 5 min between series & 10 min between groups.
Salgado et al. (2015)	14	m	26 ± 5	PRO Football	☐	☑	☑	☐	40-60	3	Fatigue	Football match 90 min.
Skinner et al. (1986)	11	m	23 ± 1	Navy SEALs	☐	☑	☑	☐	05-25	5	Fatigue	3 miles sprinting (alternate 1 & 1/4 mile all-out; 90 s rest) & 2x treadmill session (2 min., 4.8-5.6 kmh^{-1}, 15%; 90s rest between sessions).
Van Tiggelen et al. (2008)	64	b	25 ± 4	healthy	☑	☑	☑	☐	45	3	Fatigue	Isokinetic MVC ($120\,°s^{-1}$, 150s, 30 rest) until fatigue (first 5 repetitions $<$50% PT_{max} assessed in the previous series.
Vila-Cha et al. (2011)	15	m	25 ± 4	healthy	☐	☑	☑	☐	120	2	Fatigue	Eccentric knee extension ($60\,°s^{-1}$, 4 sets, 25 reps, 3 min rest).
									150	2	Fatigue	Eccentric knee extension ($60\,°s^{-1}$, 4 sets, 25 reps, 3 min rest).
Wieber et al. (2023)	25	b	25 ± 2	Sport students	☑	☑	☑	☑	random	5	BP & DoM	

BP=body position; *b*=both genders; *DoM*=direction of Movement; *f*=female; *m*=male; *META*=meta-analysis allocation; *Mov.*=Movement *MVC*=maximal voluntary contraction *PRO*=professional athlete; *PT*=peak torque; *reps*=repetitions.
