# Supplementary material for: Factors that influence the angular error in active knee angle reproduction tests: A systematic review and meta‐analysis
Source: J Exp Orthop. 2024 Jul 24;11(3):e12091. doi: 10.1002/jeo2.12091 (PMC11269366; doi:10.1002/jeo2.12091)
Supplement: Supplementary file 5 — Supporting information. [file JEO2-11-e12091-s004.docx]

**Supplementary material 1**

**Table** Search strategy keywords and MeSH terms for the five data repositories.

| Data repository | Keywords | # |
| --- | --- | --- |
| PubMed | (((knee OR ”knee joint” OR (knee[MeSH Terms]) OR ”knee-joint” AND (reproduct* OR propriocept* OR ”joint position sense” OR ”position sense” OR (proprioception[MeSH Terms])) AND (”angle” OR”error”)))) | 664 |
| Web of Science | ((ALL=(knee OR ”knee joint” OR ”kneejoint”)) AND ALL=(propriocept* OR ”position sense” OR reproduct* OR ”joint position sense”)) AND ALL=(error OR angle) | 765 |
| IEEE | (knee OR ”knee-joint” OR ”knee joint”) AND (propriocept* OR reproduct* OR  ”position sense” OR ”joint position sense”)  AND (angle OR error) | 38 |
| Cochrane | knee OR ”knee-joint” OR ”knee joint”) AND (reproduct* OR ”joint position sense”  OR propriocept* OR ”position sense”) AND  (”error” or ”angle”) | 244 |
| SPORTDiscus | TX ( knee OR ”knee joint” OR ”knee-joint”)  AND TX ( propriocept* OR ”position sense” OR reproduct* OR ”joint position sense” )  AND TX (error* OR angle*) | 312 |
| Overall |  | 2023 |

*IEEE*: Institute of Electrical and Electronics Engineers library.
